# Effects of coffee on driving performance during prolonged simulated highway driving

**DOI:** 10.1007/s00213-012-2647-7

**Published:** 2012-02-08

**Authors:** M. A. J. Mets, D. Baas, I. van Boven, B. Olivier, J. C. Verster

**Affiliations:** Division of Pharmacology, Utrecht Institute for Pharmaceutical Sciences, Utrecht University, Universiteitsweg 99, 3584 CG Utrecht, The Netherlands

**Keywords:** Caffeine, Automobile driving, Fatigue, Sleepiness

## Abstract

**Rationale:**

Coffee is often consumed to counteract driver sleepiness. There is limited information on the effects of a single low dose of coffee on prolonged highway driving in non-sleep deprived individuals.

**Objectives:**

The aim of this study was to examine the effects of a single cup of coffee (80 mg caffeine) on simulated highway driving performance.

**Methods:**

Non-sleep deprived healthy volunteers (*n* = 24) participated in a double-blind, placebo-controlled, crossover study. After 2 h of monotonous highway driving, subjects received caffeinated or decaffeinated coffee during a 15-min break before continuing driving for another 2 h. The primary outcome measure was the standard deviation of lateral position (SDLP), reflecting the weaving of the car. Secondary outcome measures were speed variability, subjective sleepiness, and subjective driving performance.

**Results:**

The results showed that caffeinated coffee significantly reduced SDLP as compared to decaffeinated coffee, both in the first (*p* = 0.024) and second hour (*p* = 0.019) after the break. Similarly, the standard deviation of speed (*p* = 0.024; *p* = 0.001), mental effort (*p* = 0.003; *p* = 0.023), and subjective sleepiness (*p* = 0.001; *p* = 0.002) were reduced in both the first and second hour after consuming caffeinated coffee. Subjective driving quality was significantly improved in the first hour after consuming caffeinated coffee (*p* = 0.004).

**Conclusions:**

These findings demonstrate a positive effect of one cup of caffeinated coffee on driving performance and subjective sleepiness during monotonous simulated highway driving.

## Introduction

Drowsy driving is an important cause of traffic accidents (Connor et al. 2002; Horne and Reyner 1995; Maycock 1996), and therefore, the development of effective countermeasures is essential. Consuming a cup of coffee is one of the most commonly used ways to combat driver sleepiness. An estimated 80% of the population consumes caffeine-containing beverages, often on a daily basis (Fredholm et al. 1999; Heckman et al. 2010). Caffeine (1,3,7-trimethylxanthine) is rapidly and completely absorbed in the body within approximately 45 min (Blanchard and Sawers 1983). It reaches its peak plasma concentration within 15 to 120 min after intake (Arnaud 1987), averaging around 30 min (O’Connell and Zurzola 1984; Blanchard and Sawers 1983). Its elimination half life is 1.5 to 9.5 h (Arnaud 1987; Bonati et al. 1982). Although additional mechanisms of action are involved, it is now believed that caffeine’s stimulant effects are exerted by antagonizing adenosine, primarily by blocking the adenosine A1 and A2A receptors. Adenosine is considered to be a mediator of sleep (Dunwiddie and Mansino 2001; Fredholm et al. 1999).

A great number of studies have demonstrated effects of caffeine on mood and performance (Childs and De Wit 2006; Christopher et al. 2005; Haskell et al. 2005; Lieberman et al. 1987; Olson et al. 2010). However, the effects are complex and depend on the specific tasks examined, dosages, subjects, and test conditions (Lorist and Tops 2003). Overall, caffeine was found to be specifically effective in restoring performance to baseline levels when individuals are in a state of low arousal, such as seen during the dip in the circadian rhythm, after sleep restriction, and in fatigued subjects (Nehlig 2010; Smith 2002). Indeed, many people consume coffee with the purpose to refresh or stay awake, for example, when driving a car (Anund et al. 2008; Vanlaar et al. 2008).

Several driving studies showed that caffeine improves performance and decreases subjective sleepiness both in driving simulators (Biggs et al. 2007; Brice and Smith 2001; De Valck and Cluydts 2001; Horne and Reyner 1996; Regina et al. 1974; Reyner and Horne 1997, 2000) and on the road (Philip et al. 2006; Sagaspe et al. 2007). Most of these studies tested sleep-restricted subjects. In addition, relatively high dosages of caffeine (100–300 mg) were examined. These studies showed that relatively high dosages of caffeine had a positive effect on driving performance and reduced driver sleepiness. In real life however, it is more likely that a driver consumes only one cup of coffee (80 mg of caffeine) during a break, before continuing driving. Up to now, the effects on driving performance of lower dosages of caffeine, e.g., a regular cup of coffee, have not been examined.

Therefore, the objective of this study was to examine the effects of one cup of coffee (80 mg caffeine) on prolonged simulated highway driving in non-sleep deprived individuals. Various traffic safety organizations advise drivers to take a 15-min break after 2 h of driving. The protocol used in the current study (2 h driving, a 15-min break with or without consuming caffeinated coffee, followed by 2 h of driving) was based on this advice.

## Materials and methods

This study was a double-blind, randomized, placebo-controlled, cross-over study. The study was conducted according to the ICH Guidelines for “Good Clinical Practice,” and the Declaration of Helsinki and its latest amendments. Written informed consent was obtained from the participants before taking part in the study. The study was approved by the Institutional Review Board; no medical ethical approval was required to conduct the study.

### Subjects

Twenty-four adult healthy volunteers (12 males and 12 females) were recruited by means of public advertisements at and around Utrecht University campus. Subjects were included if they were healthy volunteers, moderate caffeine drinkers (two to four cups a day), non-smokers, had a body mass index between 21 and 30, possessed a valid driver’s license for at least 3 years, and drove more than 5,000 km per year.

Sleep disturbances were assessed with the SLEEP-50 questionnaire (Spoormaker et al. 2005), and excessive daytime sleepiness was examined using the Epworth Sleepiness Scale (ESS; Johns 1991), filled out by participants on the screening day.

Before the start of each test day, urine samples were collected to test for drugs of abuse including amphetamines (including MDMA), barbiturates, cannabinoids, benzodiazepines, cocaine, and opiates (Instant-View, Alfa Scientific Designs Inc.) and pregnancy in female subjects (β-HCG test). To test for the presence of alcohol, the Dräger Alcotest 7410 Breath Analyzer was used. From 24 h before the start of the test day until completion of the test day, alcohol consumption was not permitted. Caffeinated beverages were not allowed from awakening on test days until the end of the tests.

### Study design

Participants were screened and familiarized with the test procedures during a training day. When meeting all inclusion and passing all exclusion criteria, subjects performed a practice session in the STISIM driving simulator and completed the Simulator Sickness Questionnaire (Kennedy et al. 1993) to identify possible simulator sickness. Included subjects were randomly assigned to a treatment order comprising decaffeinated coffee and caffeinated coffee (80 mg) administered during a break.

Upon arrival, possible use of drugs or alcohol, pregnancy, illness, and medication were checked. In addition, quality and duration of sleep was assessed using the 14-item Groningen Sleep Quality Scale (Mulder-Hajonides van der Meulen et al. 1980). When all criteria were met, a 120-min drive in the STISIM driving simulator was conducted. Thereafter, a 15-min break was scheduled in which subjects received the double-blind treatment. After the break, another 120-min driving session was performed. Every hour, subjective assessments of driving quality, driving style, mental effort to perform the test, and sleepiness were conducted. Test sessions were scheduled at the same time for each subject, either in the morning (0800–1300 hours) or in the afternoon (1300–1700 hours).

### Treatments

This study aimed to mimic the effect of a cup of coffee drivers consume when having a break along the highway. Treatments were 2.68 g of Nescafé Gold® instant coffee containing 80 mg caffeine or 2.68 g of Nescafé Gold® decaffeinated coffee dissolved in 180 ml boiled water. To confirm that each cup of coffee contained 80 mg of caffeine, the amount of caffeine in the instant coffee was determined with high-performance liquid chromatography (HPLC; Shimadzu LC-10AT VP equipped with UV–Vis detector). The column was a reversed-phase Select B column Lichrocart HPLC C18, 5 μm, length, 0.125 m, Ø = 4.6 mm. All of the procedures were carried out isocratic. The separation was done at room temperature. Caffeine and the spiked matrices were separated with a mobile phase of 20% MeOH and 10 mM HClO_4_, at a flow rate of 0.5 mL/min. The injection volume was 5 μL, and the detection was carried out at 273 nm. The mean (SD) amount of caffeine per gram Nescafé Goud instant coffee samples (*n* = 10) was 29.79 (0.656) mg/g. The mean amount of caffeine in decaffeinated coffee was 0.79 mg/g. The accuracy of determinations was 98.1% (SD, 0.56). Because both the precision and the accuracy met up to the requirement demands, all of the results of this HPLC determination can be concluded with certainty.

Treatments were administered double-blind, and a nose clip was worn to enhance treatment blinding. Drinks were consumed within 5 min, starting from 5 min after onset of the break.

### STISIM highway driving test

Driving tests were performed in a fixed-base driving simulator employing STISIM Drive™ (version M300, Systems Technology Inc., Hawthorne, CA, USA). This is an interactive system in which the roadway scenery is projected on a screen (2.10 × 1.58 m), 1.90 m in front of the center of the steering wheel of the car unit (Mets et al. 2011a). The 100-km highway driving test scenarios were developed (EyeOctopus BV) in accordance with Dutch traffic situations, including a two-lane highway in each direction and a monotonous environment with trees, occasional hills and bridges, and other traffic. The duration of each 100-km scenario is approximately 60 min. Two scenarios (200 km) were conducted before a 15-min break, and two other scenarios (200 km) thereafter (Mets et al. 2011a).

Subjects were instructed to drive with a steady lateral position within the right, slower, traffic lane with a constant speed of 95 km/h. Overtaking slower-moving vehicles was allowed. During blinded editing, these maneuvers were removed from the data, before statistical analysis of the “clean” data. The primary outcome variable was the standard deviation of lateral position (SDLP, centimeters), expressing the weaving of the car (Verster and Roth 2011). The standard deviation of speed (SDS, kilometers per hour) was the secondary outcome measure. Mean speed (MS, kilometers per hour) and mean lateral position (MLP, cm) were control variables.

### Subjective assessments

After each hour of driving, questionnaires were administered on subjective sleepiness and driving performance. Subjective sleepiness was measured by means of the Karolinska Sleepiness Scale (KSS), ranging from 1 (very alert) to 9 (very sleepy, fighting sleep) (Åkerstedt and Gillberg 1990).

Driving task-related questionnaires comprised mental effort to perform the driving test (Meijman et al. 1986; Zijlstra and Van Doorn 1985), subjective driving quality, and driving style (McCormick et al. 1987). Completing the questionnaires took approximately 2 min, after which, the driving task was immediately resumed.

### Statistical analysis

Statistical analyses were performed with SPSS, version 19. For each variable, mean (SD) was computed for each subsequent hour. Data of the first 2 h were compared, to confirm that no significant differences between the treatment days were present before the break and treatment administration. To determine whether caffeinated coffee has an effect on driving performance, data from the third and fourth hour were compared using a general linear model for repeated measures (two-tailed, *p* ≤ 0.05).

## Results

A total of 24 subjects (12 males and 12 females) completed the study. Their mean (SD) age was 23.2 (1.6) years old; on average, they consumed 2.5 (0.7) caffeinated drinks per day, had a mean (SD) body mass index of 23.9 (2.7), possessed a valid driver’s license for 58.8 (17.9) months, and on average drove 12,979 (SD, 10,785) km per year. All subjects reported normal sleep quality and duration on the nights before the test days with no differences observed between the two test conditions. Results from the study are summarized in Table 1. There were no significant order effects (caffeinated–decaffeinated coffee versus decaffeinated–caffeinated coffee) or time-of-testing effects (a.m. versus p.m.).

**Table 1:** Effects of caffeinated coffee in comparison to decaf on simulated driving performance and subjective sleepiness

	Time	Decaffeinated coffee	Caffeinated coffee
Driving test results			
Standard deviation of lateral position (cm)	1	21.43 (4.37)	22.11 (3.67)
	2	23.65 (5.90)	24.13 (4.76)
	3	22.92 (4.61)	21.08 (3.74)*
	4	23.69 (4.72)	22.41 (4.37)*
Standard deviation of speed (km/h)	1	0.85 (0.44)	0.88 (0.35)
	2	0.98 (0.51)	1.1 (0.61)
	3	1.03 (0.72)	0.78 (0.34)*
	4	1.15 (0.77)	0.87 (0.56)*
Mean lateral position (cm)	1	−18.04 (12.71)	−18.03 (10.47)
	2	−19.24 (12.60)	−18.98 (9.98)
	3	−18.63 (12.31)	−20.16 (11.05)
	4	−18.17 (11.54)	−18.93 (10.80)
Mean speed (km/h)	1	95.40 (0.19)	95.42 (0.21)
	2	95.46 (0.16)	95.40 (0.26)
	3	95.44 (0.31)	95.54 (0.18)
	4	95.53 (0.15)	95.54 (0.25)
Subjective driving assessments			
Driving quality	1	9.75 (3.66)	9.08 (4.10)
	2	9.01 (2.81)	8.48 (3.46)
	3	9.70 (3.89)	11.84 (2.82)*
	4	9.23 (3.02)	10.60 (3.41)
Mental effort	1	5.33 (2.30)	5.70 (2.47)
	2	5.84 (2.76)	6.39 (2.50)
	3	5.89 (2.82)	4.50 (2.36)*
	4	5.72 (2.38)	4.90 (2.93)*
Subjective sleepiness scores			
Karolinska sleepiness scale	Baseline	3.25 (0.94)	3.33 (0.87)
	1	6.08 (1.67)	5.83 (2.16)
	2	6.17 (1.95)	6.29 (1.97)
	3	6.13 (2.11)	4.21 (1.47)*
	4	5.79 (1.59)	4.54 (1.86)*

Mean (SD) is shown for each parameter. Driving quality ranges from 0 (“I drove exceptionally poorly”) to 20 (“I drove exceptionally well”). For mental effort, higher scores indicate higher effort; higher KSS scores indicate increased subjective sleepiness

*p < 0.05 compared to decaf

### Driving test

**Fig. 1:**
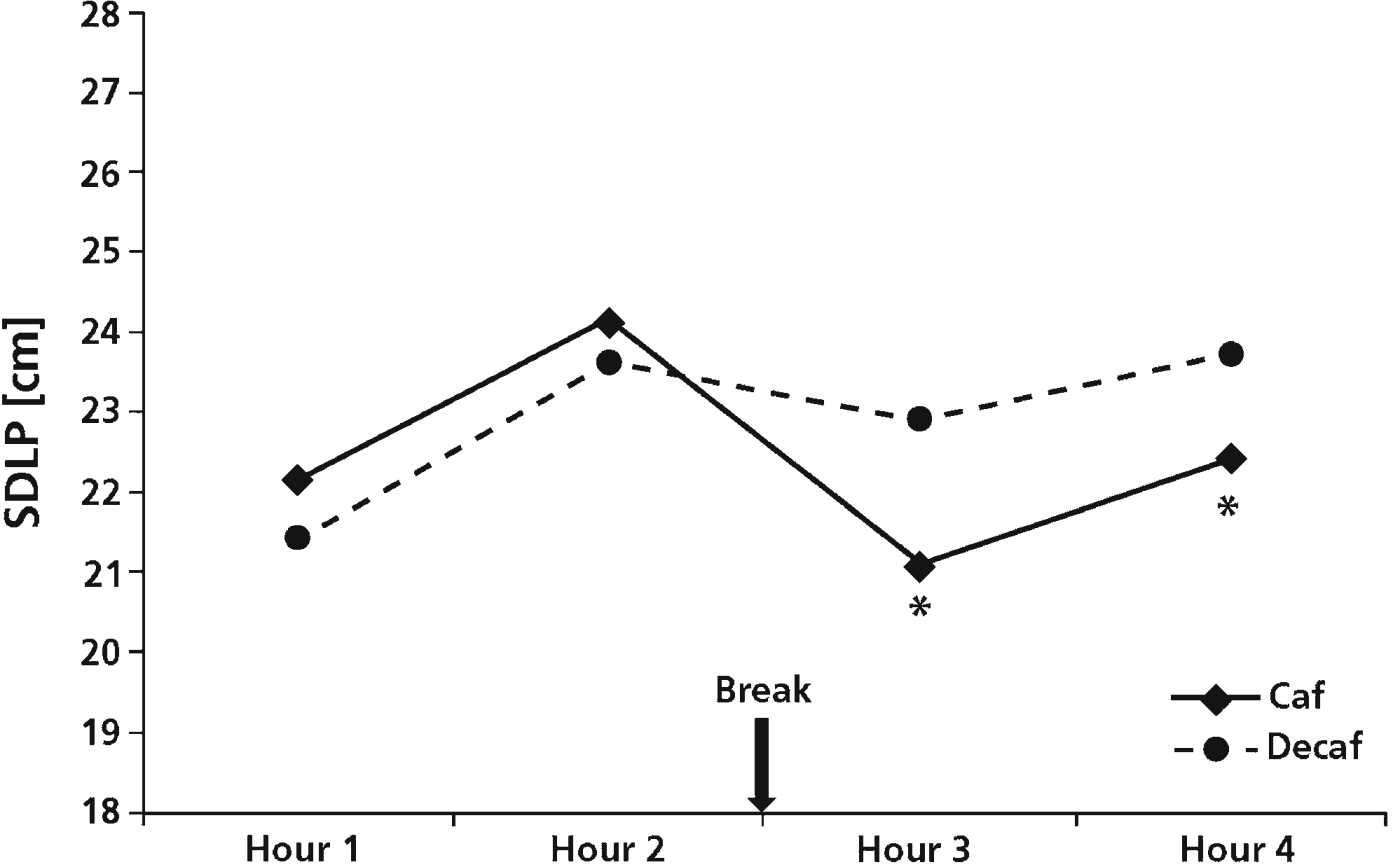
Standard deviation of lateral position (SDLP). *Asterisks* indicate significant difference compared to placebo (*p* < 0.05)

Figure 1 shows the effect of caffeinated coffee consumption on driving performance. No significant differences in SDLP were observed before the break. However, both in the first (*F*_(1,23)_ = 5.8; *p* = 0.024) and in the second hour (*F*_(1,23)_ = 6.4; *p* = 0.019) after the break, caffeinated coffee significantly reduced SDLP.

**Fig. 2:**
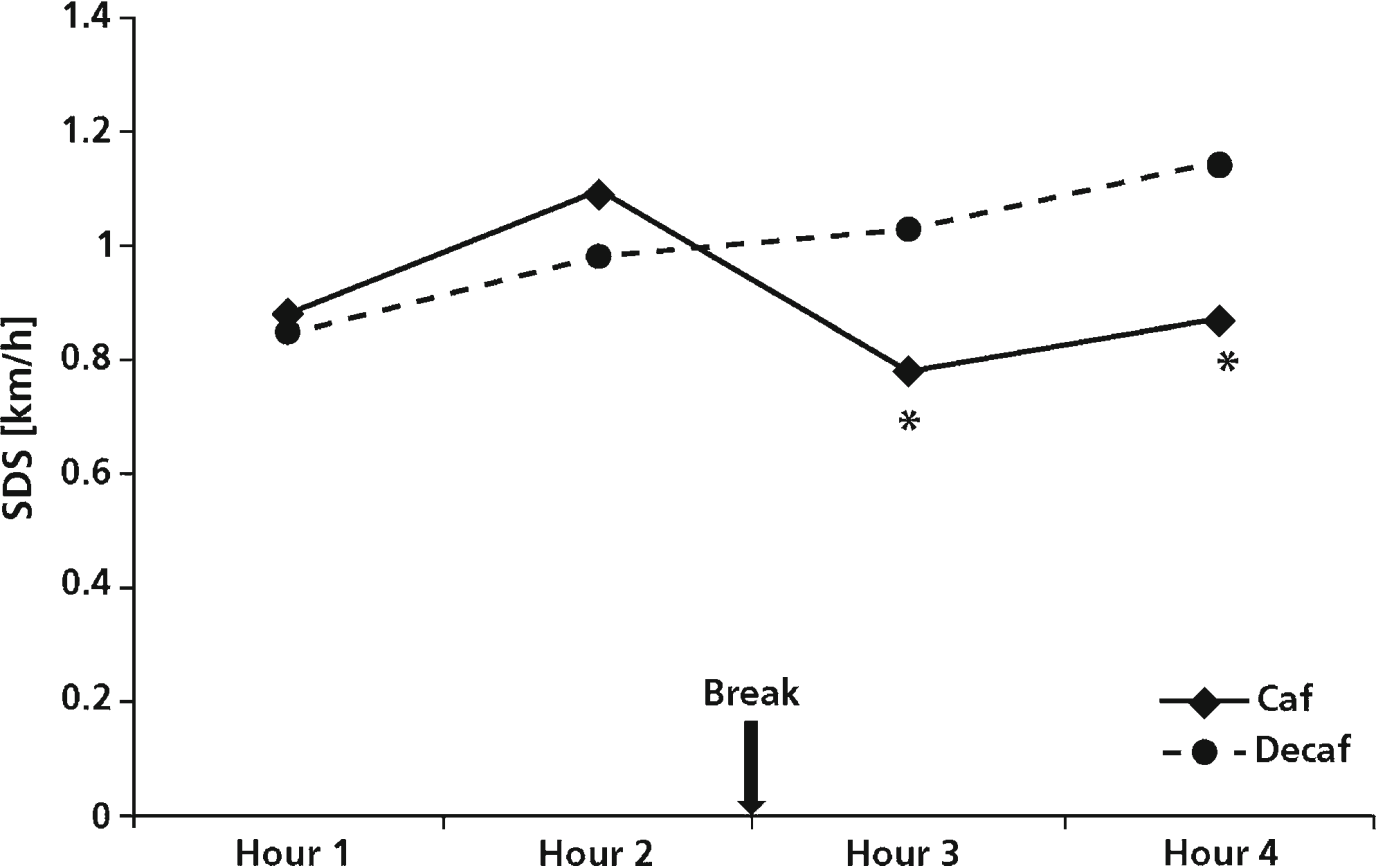
Standard deviation of speed (SDS). *Asterisks* indicate significant difference compared to placebo (*p* < 0.05)

In line, caffeinated coffee significantly reduced SD speed in the third (*F*_(1,23)_ = 5.8; *p* = 0.024) and fourth hour (*F*_(1,23)_ = 13.0; *p* = 0.001) of driving (see Fig. 2).

No effects were found on mean speed or mean lateral position, confirming that subjects performed the test according to the instructions.

### Subjective driving assessments

Compared to decaffeinated coffee, caffeinated coffee improved subjective driving quality in the third hour of driving (*F*_(1,23)_ = 10.5; *p* = 0.004), but not in the fourth (*F*_(1,23)_ = 2.6; *p* = n.s.). Subjects indicated that the mental effort needed to perform the test after caffeinated coffee was significantly reduced in the third (*F*_(1,23)_ = 11.4; *p* = 0.003) and fourth hour of driving (*F*_(1,23)_ = 5.9; *p* = 0.023). In addition, drivers rated their driving quality as significantly more considerate, responsible, and safer in the caffeinated coffee condition (see Table 1).

### Subjective sleepiness

**Fig. 3:**
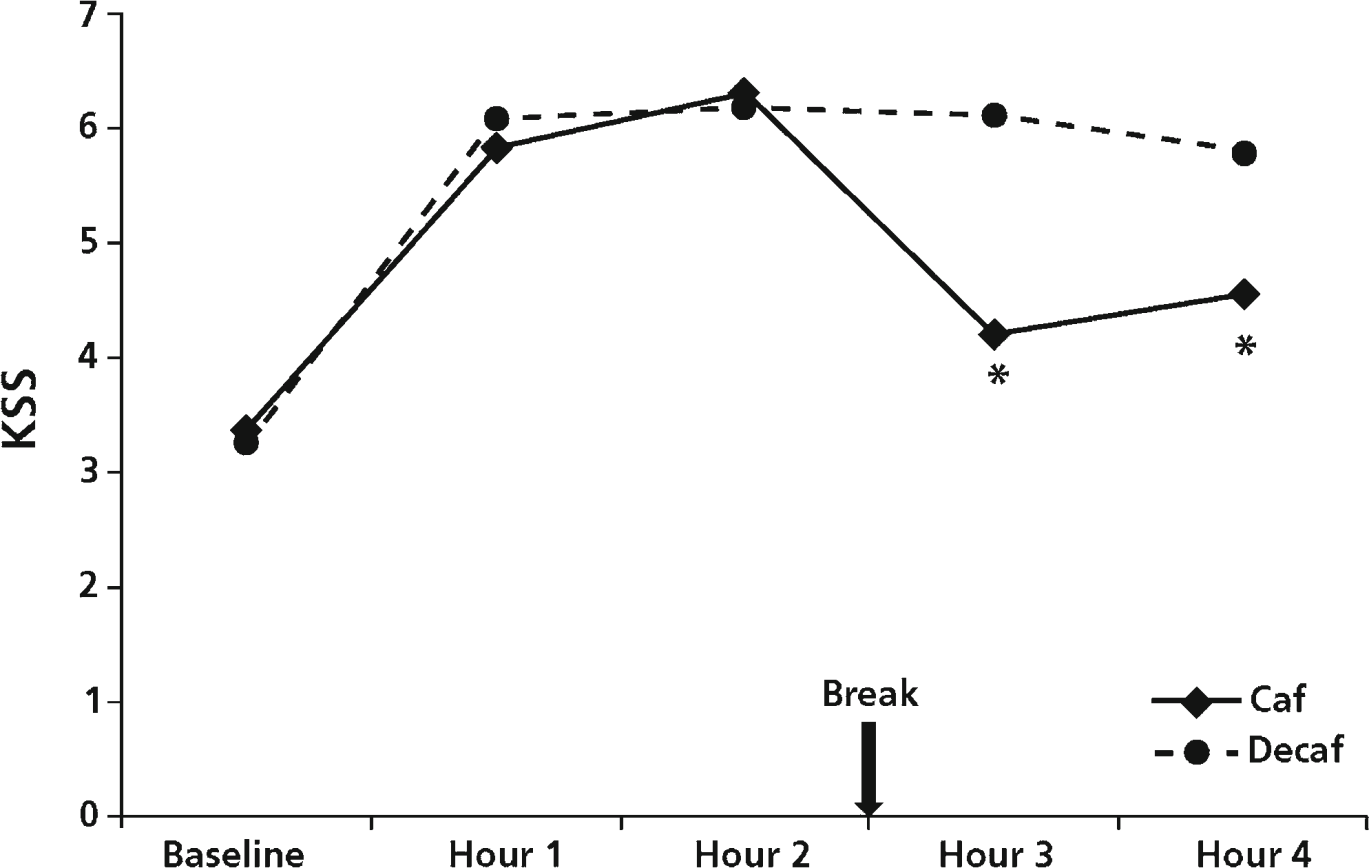
Karolinska sleepiness scale. *Asterisks* indicate significant difference compared to placebo (*p* < 0.05)

After the break with caffeinated coffee, drivers reported significantly lower sleepiness scores as compared to the break with decaffeinated coffee. This effect was significant both in the third (*F*_(1,23)_ = 18.5; *p* < 0.001) and the fourth hour of driving (*F*_(1,23)_ = 11.9; *p* = 0.002) (see Fig. 3).

## Discussion

This study demonstrates that one cup of caffeinated coffee (80 mg caffeine) significantly improves driving performance and reduces driver sleepiness.

Both lane keeping (SDLP) and speed maintenance were improved up to 2 h after caffeine consumption. The effect on SDLP of caffeinated coffee, compared to placebo, is comparable to changes observed with a blood alcohol concentration of 0.05% (Mets et al. 2011b), i.e., the legal limit for driving in many countries, but in the opposite direction. Hence, the improvement by caffeinated coffee can be regarded as clinically relevant.

The magnitude of driving improvement observed after coffee consumption was comparable to the improvement seen in a driving study with Red Bull® Energy Drink using the same design and driving test (Mets et al. 2011a), and on-road driving studies showing improvement after administration of methylphenidate to patients with attention-deficit hyperactivity disorder (Verster et al. 2008).

The improvement in objective performance was accompanied by improvement in subjective assessments of sleepiness and driving performance. An average decrease of almost 2 points (out of 7) on the KSS scale was observed after the intake of caffeinated coffee as compared to decaffeinated coffee. The average KSS score was 6 (“some signs of sleepiness”) in the decaffeinated coffee condition compared to 4 (“rather alert”) in the caffeinated coffee condition. These findings are in agreement with the pharmacokinetic profile of caffeine (Tmax ≈ 30 min; T1/2 > 2 h), as well as with the known actions of caffeine as a sleepiness countermeasure with the ability to restore performance to baseline.

Up to now, higher dosages of caffeine (150–250 mg, comparable to two to three cups of regular coffee) have been shown to be effective in counteracting sleep restriction (<5 h spent in bed) when driving in the early morning (Reyner and Horne 2000) and in the early afternoon (Horne and Reyner 1996; Reyner and Horne 1997). A moderate caffeine dosage (100 mg) decreased drifting out of lane and reduced subjective sleepiness in drivers who had slept for no more than 4 h (Biggs et al. 2007). Furthermore, caffeine (3 mg/kg, approximately 225 mg in a 75-kg adult) improved steering accuracy in non-fatigued volunteers (Brice and Smith 2001). Slow release caffeine capsules (300 mg) had similar effects (De Valck and Cluydts 2001). Interestingly, caffeine decreased lane drifting both in individuals who had spent 4.5 h in bed and in those who had spent 7.5 h in bed, while effects on speed maintenance, fatigue, and sleepiness were only observed after 4.5 h spent in bed (De Valck and Cluydts 2001). Two on-the-road driving studies on a public highway in France confirmed these findings and showed that relatively high dosages of caffeine (200 mg) improved nighttime driving both in young and in middle-aged drivers (Philip et al. 2006; Sagaspe et al. 2007).

The current results are in agreement with these studies, but further show that lower caffeine content found in one regular cup of coffee also significantly improves driving performance and reduces driver sleepiness. In addition, where studies find effects on lane keeping, the current study shows that speed maintenance is also affected, indicating more pronounced effects on vehicle control. The importance of this finding is evident, since it can be assumed that in order to refresh, most drivers consume only one cup of coffee during a break, instead of three or four. Driving simulator research has several limitations, which are discussed elsewhere (e.g., Mets et al 2011b; Verster and Roth 2011, 2012). Therefore, it is important to replicate and confirm our findings in an on-the-road driving study. Furthermore, subjects who were tested in the afternoon may have had an additional effect of the afternoon dip in the circadian rhythm. For this reason, each subject had his test days at the same time of day. However, no significant differences were found between subjects tested in the morning and those tested in the afternoon. Further studies could examine if a low dose of caffeine has similar effects on (professional) drivers who are sleep-restricted or shifted their day–night rhythm, since current studies have only been performed with higher dosages of caffeine.

In conclusion, the present study demonstrates that one cup of caffeinated coffee (80 mg caffeine) has a positive effect on continuous highway driving in non-sleep restricted, healthy volunteers.
